# Veterinary Medical Society of the University of Pennsylvania

**Published:** 1890-05

**Authors:** B. F. Senseman

**Affiliations:** Secretary


*Veterinary Medical Society of the University of Pennsylvania.*—This society held a meeting at the Veterinary Department April 16th, 1890. Mr. Landes presided. At roll call eighteen members responded. The minutes of the previous meeting were read and approved. The executive committee had nothing to report.

The following amendment to the Constitution was offered, and will be voted upon at next meeting:

Article XI, Sec. III.—"Any member in arrears to the amount of $2.00 shall be notified by the secretary, and if the amount of arrears is not paid at the next regular meeting he shall be expelled."

Mr. John Harrigan, Class of '90, was elected an active member of the society. Mr. Kean reported a case illustrating why a veterinarian should always carry his blacksmith tools with him. An animal, supposed to be foundered, upon examination revealed suppurating corns on heels of both forefeet; also, case of epilepsy found on the street, and a case of leucorrhœa. Mr. Eshleman reported a case of abortion supposed to be due to copulation. Mr. Meisner reported a case of azoturia in which the animal remained paralyzed for six weeks, and recovered. Mr. Pearson read a paper on "El Dourine," and referred especially to the outbreak in the United States and to the differences existing between the symptoms presented in the Illinois outbreak and those described by European writers. He reviewed the history of the introduction and suppression of the disease in this country in detail.

B. F. SENSEMAN, *Secretary.*

